# Psychotic symptoms during puerperium. Regarding a clinical case

**DOI:** 10.1192/j.eurpsy.2025.1845

**Published:** 2025-08-26

**Authors:** C. Mateo Garcia, Á. Blanco Niño, C. Pérez Puente, I. Esteban Hernández

**Affiliations:** 1 Hospital Virgen del Puerto, Plasencia, Spain

## Abstract

**Introduction:**

The puerperium, or postpartum period, begins immediately after childbirth and is typically considered to last for six to eight weeks. During this time, there is an increased risk of psychiatric pathology, particularly psychotic and affective disorders, due to various factors such as the heightened stress of caring for a newborn and hormonal changes.

**Objectives:**

To analyze the different diagnostic options for a patient presenting with acute psychotic symptoms during the postpartum period, including those not recognized by the DSM-V.

**Methods:**

Presentation of a patient’s case and a review of the existing literature concerning the possible diagnoses that were considered.

**Results:**

We present the case of a 31-year-old female with a history of allergy to beta-lactam antibiotics as her only significant medical history, and a personal psychiatric history of anorexia nervosa (currently resolved) and bipolar disorder. The bipolar disorder had been treated with valproic acid and olanzapine since 2015, medications she discontinued upon learning of her pregnancy. She gave birth to a baby boy four days before seeking care in the emergency department. On the same morning of the consultation, her baby was diagnosed with a renoureteral malformation and prescribed amoxicillin treatment. The patient presented to the emergency department with very acute psychotic symptoms that had started that same day, notably a very disorganized speech and delusional ideas of harm, expressing the belief that her family was poisoning her with the prescribed amoxicillin. During her admission to the acute psychiatry ward, her condition evolved rapidly and very favorably, in around 48 hours and she was treated with olanzapine. Several diagnostic possibilities were considered, including postpartum psychosis, brief psychotic episode, manic episode, or even an organic cause. The rapid onset and therapeutic response ruled out the diagnosis of a manic episode. No organic cause was found to explain the symptoms. It also did not meet the temporal criteria required for a postpartum psychosis. Finally, the diagnosis was brief psychotic episode, triggered by an acute stressor.

**Conclusions:**

When managing a patient in the postpartum period, it is crucial to have a comprehensive understanding of the various diagnostic possibilities, including those not covered by the DSM-V or those that may have a somatic cause, in order to provide appropriate and holistic care.

**Disclosure of Interest:**

None Declared

